# MDM2 as a Rational Target for Intervention in CDK4/6 Inhibitor Resistant, Hormone Receptor Positive Breast Cancer

**DOI:** 10.3389/fonc.2021.777867

**Published:** 2021-11-03

**Authors:** Neil Portman, Julia Chen, Elgene Lim

**Affiliations:** ^1^Cancer Theme, Garvan Institute of Medical Research, Darlinghurst, NSW, Australia; ^2^ St. Vincent’s Clinical School, University of New South Wales (UNSW) Sydney, Kensington, NSW, Australia

**Keywords:** breast cancer, MDM2, CDK4/6 inhibitor, estrogen receptor, endocrine resistance

## Abstract

With the adoption of inhibitors of cyclin dependent kinases 4 and 6 (CDK4/6i) in combination with endocrine therapy as standard of care for the treatment of advanced and metastatic estrogen receptor positive (ER+) breast cancer, the search is now on for novel therapeutic options to manage the disease after the inevitable development of resistance to CDK4/6i. In this review we will consider the integral role that the p53/MDM2 axis plays in the interactions between CDK4/6, ERα, and inhibitors of these molecules, the current preclinical evidence for the efficacy of MDM2 inhibitors in ER+ breast cancer, and discuss the possibility of targeting the p53/MDM2 *via* inhibition of MDM2 in the CDK4/6i resistance setting.

## Introduction

The widespread adoption of inhibitors of cyclin dependent kinases 4 and 6 (CDK4/6i) for the treatment of advanced and metastatic estrogen receptor positive (ER+) breast cancer will fundamentally change the biology and natural history of a disease that effects a significant proportion of the population. In the advanced setting, resistance to treatment is almost inevitable, either *via* underlying mechanisms or by the acquisition of resistant phenotypes. This leads to an urgent unmet clinical need for novel therapeutics in the CDK4/6i resistant setting; a setting that crosses multiple checkpoints and signalling pathways involved in cell cycle progression, senescence and apoptosis. In this review we will consider the integral role that the p53/MDM2 axis plays in the interactions between CDK4/6, ERα, and inhibitors of these molecules, the current preclinical evidence for the efficacy of MDM2 inhibitors in ER+ breast cancer, and discuss the possible outcomes of targeting p53/MDM2 in the CDK4/6i resistance setting.

There are currently three CDK4/6i approved for clinical use, including palbociclib and ribociclib which are similar in terms of mechanism of action and target affinity, and are used clinically in combination with endocrine therapy; and abemaciclib, which has a slightly different affinity profile and has additional activity against other CDKs, particularly CDK9 (1). Abemaciclib is currently the only CDK4/6i approved for use as a single agent and in combination with endocrine therapy. Inhibition of CDK4/6 delivers therapeutic action by blocking phosphorylation of the key cell cycle regulator retinoblastoma protein (pRb) early in the G1 phase of the cell cycle (2). Hypophosphorylated pRb interacts with and inactivates transcription factors in the E2 promoter binding factor (E2F) family which are responsible for initiating the cell cycle process in G1 leading to S phase (3). CDKs operate in complex with cyclins which regulate and direct the kinase activity. In the case of CDK4/6 these are the D type cyclins (4, 5) which are transcriptional targets of ERα (6) and frequently upregulated in ER+ breast cancer (7), providing the rationale for targeting this axis in ER+ breast cancer. Unfortunately, the advanced stage of disease in which CDK4/6i are currently indicated is considered to be incurable, with treatment intent aimed at disease management and the development of resistance to treatment being inevitable. Indeed, despite the clear effectiveness of CDK4/6i demonstrated in clinical trials with an almost doubling progression free survival (8–10), resistance is poised to become a major challenge for the management of advanced ER+ breast cancer in the developed world as it is expected that all patients in this setting will receive CDK4/6i as part of their treatment.

## Resistance to CDK4/6i in ER+ Breast Cancer

With the majority of patients with advanced ER+ breast cancer expected to receive CDK4/6i as part of their treatment, and the high likelihood of the development of resistance to CDK4/6i in a given patient, the post CDK4/6i treated phenotypes represent new biological states for ER+ breast cancer (11). A number of mechanisms of CDK4/6i resistance have been proposed or identified in preclinical models and clinical samples, the diversity of upstream mechanisms reflecting the integral position of cell cycle control in the functioning of the cell. Detailed mechanisms of resistance to CDK4/6i in breast cancer have been the subject of several recent reviews (11–15), but as a brief summary, current evidence largely points towards a unifying theme of eliminating hypophosphorylated pRb to allow progression through the G1 cell cycle checkpoint. In clinical samples, *RB1* mutation or deletion have been reported as direct mechanisms to eliminate functional pRb (16). Most other proposed or identified mechanisms involve means by which hyperphosphorylation of pRb can be restored. Mutation of the FAT Atypical Cadherin 1 (*FAT1*) gene has also been identified at a small percentage of clinical samples (17), leading to dysregulation of the HIPPO pathway through the accumulation of key HIPPO pathway components and increased expression of CDK6. Other proposed mechanisms of CDK4/6i resistance include 1) upregulation of cyclin D isoforms (18–20), which may underlie the relative ineffectiveness of palbociclib and ribociclib as a single agent in the absence of an endocrine therapy backbone as ERα is still free to promote expression of cyclin D; 2) dysregulated early phosphorylation of pRb by CDK2 (21, 22); 3) upregulation of proliferative signalling leading to pRb hyperphosphorylation *via* dysregulation of the phosphatidylinositol 3 kinase (PI3K) and mitogen activated protein kinase (MAPK) pathways, for example *via* upregulation of FGFR signalling (19, 20, 23, 24); and 4) deregulation of immune associated pathways, for example increased interferon α and interferon γ signalling that has been linked to reduced sensitivity and resistance and to CDK4/6i in clinical samples and preclinical models (25–27).

Many of these mechanisms of resistance are targetable using current drugs or drugs in development. Inhibitors of the PI3K pathway and MAPK pathways are already in clinical use and drugs targeting CDK2, and CDK4/6i with higher affinity and/or specificity are currently in development. However, alongside the cell cycle control axis of pRb, a second major pathway operates in the form of the p53/MDM2 axis. The p53/MDM2 axis works both through and around the pRb axis to control cell cycle progression and entry into states such as quiescence, senescence, and apoptosis. Importantly, this pathway can be targeted *via* MDM2 to activate tumour suppressive outcomes that may be able to bypass diverse mechanisms of resistance to CDK4/6i altogether.

## MDM2: A Nexus of Proliferative and Anti-Proliferative Signalling

MDM2, along with its paralogue MDM4, is most well known as the major negative regulator of p53. MDM2 and MDM4 form homo- and heterodimers to interact with p53 and block its transcriptional activity. MDM2 has additional functionality as an E3 ubiquitin ligase, targeting p53 and other substrates, including itself, for degradation *via* the proteasome. p53 becomes activated in response to genotoxic or oncogenic stress *via* post-translational modifications that inhibit the interaction with MDM2; stabilising p53 and initiating a transcriptional programme involving both upregulation and downregulation of transcripts to induce cell cycle arrest, senescence, or apoptosis. MDM2 is itself a transcriptional target of p53, such that activation of p53 results in increased expression of MDM2 thus establishing a negative feedback loop to reign in p53 once it is no longer required. MDM2 autoubiquitination occurs whilst DNA damage signals persist (28), supporting the post-translational modifications of p53 to remain active despite increased production of MDM2.

MDM2 is a particularly attractive target for intervention in the setting of ER+ breast cancer because a therapeutically relevant activation of p53 generally requires p53 to be wildtype. Although *p53* is the most commonly mutated gene across all breast cancer, in the ER+ setting, mutation rates are relatively low at 20%-25% (29). However, the relevance of MDM2 to the ER+ phenotype goes much deeper. Although the *MDM2* gene is rarely amplified in ER+ breast cancer, relatively high levels of MDM2 protein are commonly observed, occurring in at least 38% of cases (30, 31). In response to its cognate ligand oestradiol (E2), ERα directly upregulates transcription of both p53 and MDM2 (32–36). Upregulation of transcription factors in the AP1 and ETs families coincide with the development of ER+ breast cancer and are able to promote increased transcription of MDM2, even in the absence of p53, from the normally p53-dependent MDM2 promoter (37). siRNA-mediated ablation of ERα (32) or treating ER+ breast cancer cells with tamoxifen (38) (an ERα modulator) or fulvestrant (33, 38) – (a pure ERα antagonist that also leads to ERα degradation) blocks the E2-dependent upregulation of MDM2.

**Figure 1:**
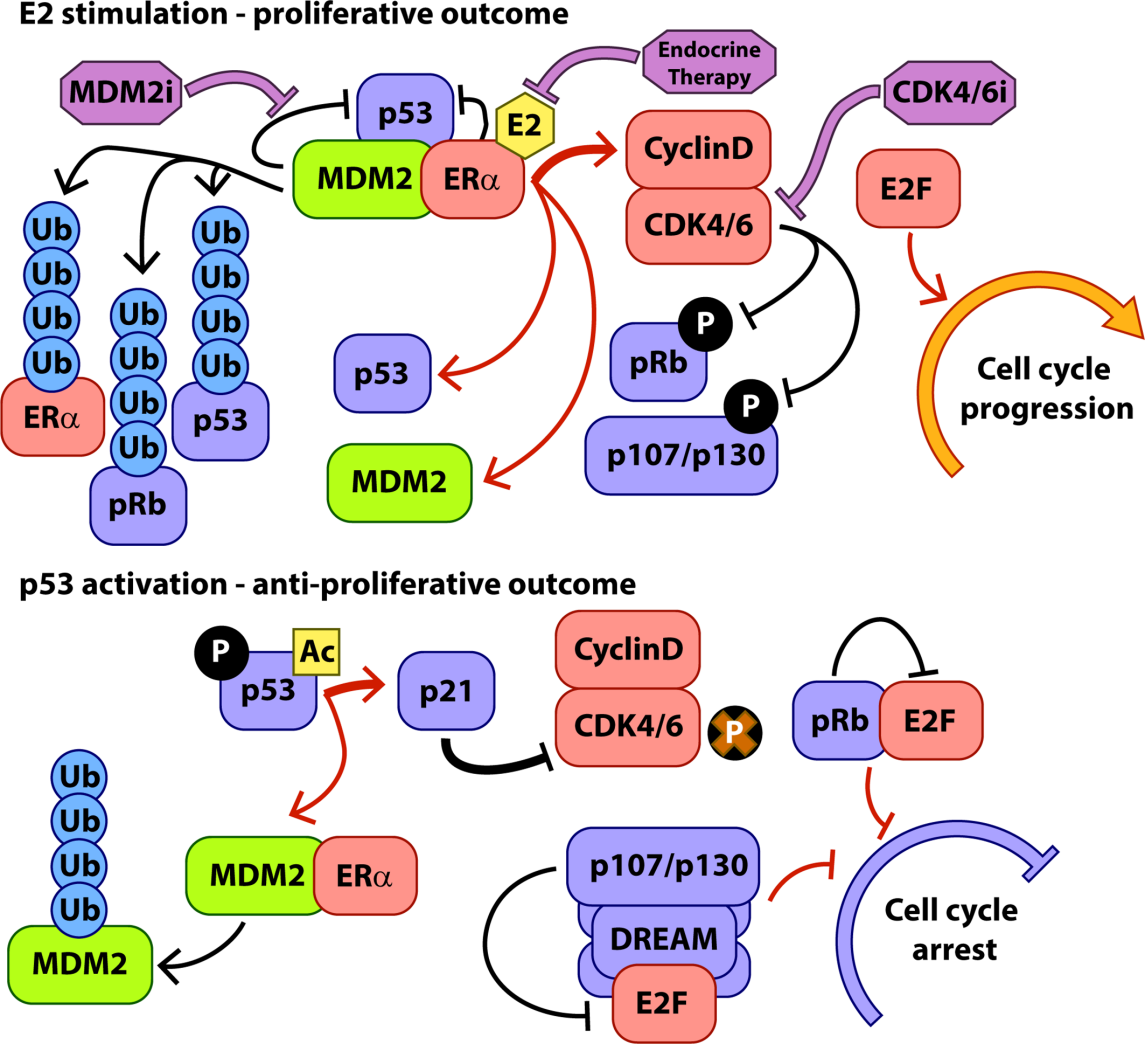
Key interactions of MDM2 during hormone-stimulated proliferation and p53 activation. Protein interactions are indicated with black arrows, transcriptional interactions with red arrows, and the activity of therapeutic interventions with purple arrows. P, phosphorylation, Ac, Acetylation, Ub, Ubiquitylation.

Genomic and proteomic interactions between p53 and ERα are well documented and MDM2 appears to play a central role in mediating the outcomes of these interactions in response to proliferative and anti-proliferative signalling inputs (**Figure 1**). ERα, p53, and MDM2 form a complex integrated signalling system controlling cell cycling and apoptosis and rooted in the normal biology of breast development that requires expansion and regression of milk producing tissues in response to hormonal signalling during parturition.

MDM2 has been shown to positively regulate ERα transcriptional activity. In MCF7 cells, overexpression of MDM2 led to increased rates of cell proliferation and increased ERα transcriptional activity in an E2-dependent manner (39, 40). MDM2 contains numerous protein:protein interaction domains and interacts with ERα at sites in both the N and C terminals, although full length protein is required to modulate ERα- transcriptional activity (39), possibly *via* interactions with the histone acetyltransferase p300 which interacts with the central domain of MDM2 (41) and is known to act as a transcriptional co-regulator of ERα (42).

This increase in available MDM2 and p53 protein forms a key control mechanism that contributes to fine-tuning of the ERα-mediated proliferative response to E2. In the presence of E2, the formation of complexes comprising ERα, p53, and MDM2 significantly increases, resulting in higher turnover of ERα protein *via* p53 dependent MDM2-mediated ubiquitylation of ERα (34). In this way, although expression levels of ERα are increased in response to E2, the p53/MDM2 complex and a series of other ubiquitin ligase complexes (43) limit the accumulation of ligand activated ERα, allowing the cell to respond more rapidly to changes in hormone stimulation. Conversely, activating p53 such as through the DNA damage response, or chemically inhibiting the interaction between MDM2 and p53 stabilises ERα, although activated p53 then further inhibits ERα transcriptional activity (34).

## Preclinical Evaluation of MDM2 Inhibitors in ER+ Breast Cancer

These complex interactions and functional feedback loops demand a degree of caution when considering the use of MDM2 inhibitors to treat ER+ breast cancer. Activation of p53 against an activated ERα background has been shown to induce novel programmes of transcriptional activity that is not associated with p53 or ERα activation alone (44, 45) and contains both proliferative and anti-proliferative signals. It is highly likely that the outcomes of combined ERα and p53 signalling in terms of proliferative or antiproliferative response will strongly depend upon the mutational, transcriptional and protein expression landscape of the particular tumour being targeted. In the clinic, ER+ breast cancer is invariably treated with ERα-directed endocrine therapy to modify or ablate ERα signalling. Several studies have now reported on the outcomes of combining endocrine therapy (usually fulvestrant) with p53 activation (*via* standard chemotherapy or MDM2 inhibitors in clinical development) in cell line and xenograft models of endocrine-sensitive and endocrine-resistant ER+ breast cancer (29, 33, 45, 46). These studies establish a clear picture in preclinical models that the combination of endocrine therapy with p53 activation is synergistic in the endocrine sensitive setting and that endocrine therapy potentiates p53 activation in the endocrine resistant setting. The MDM2 inhibitors MI-77301 and NVP-CGM097 were tested against patient derived models of *p53* wild type, fulvestrant-resistant ER+ breast cancer by Lu and colleagues (46) and by us (29) respectively. Both inhibiters occupy the p53 binding domain of MDM2, resulting in stabilisation and activation of p53. In each case, MDM2 inhibition resulted in reduced growth in *in vitro* models of fulvestrant resistance *via* apoptosis, cell cycle arrest and senescence. Three *in vivo* models were tested across the two studies and each showed decreased growth rates with MDM2i, although the WHIM9 and WHIM18 models used by Lu et al. and treated with MI-77301 also demonstrated tumour regression which we did not observe in our Gar15-13D model treated with NVP-CGM097. All three models were immune to growth repression by fulvestrant alone and the combination of fulvestrant and MI-77301 had no additional effect on the WHIM9 model. However, both the Gar15-13D treated with NVP-CGM097 plus fulvestrant, and the WHIM18 treated with MI-77301 and fulvestrant, showed a significant and intriguing effect whereby tumour growth was significantly inhibited for an extended period beyond the cessation of treatment. Using *in vitro* models of fulvestrant resistance we were able to show that the combination of fulvestrant and MDM2i in this setting increases the rates of cell cycle arrest and senescence, but not apoptosis compared to MDM2i alone. This finding is consistent with the extended benefit seen in *in vivo* models in both studies whereby sequestering an increased proportion of cells in a senescent state could delay tumour growth after treatment had ended. It is unclear whether the differences in response in the two studies reflect different drug efficiencies or differences in the underlying biology of the models, but the similarities across the two studies, particularly in the outcome of combination therapy in the WHIM18 and Gar15-13D models, suggest that underlying tumour biology plays a significant part.

MDM2i elicit a p53 response in all models of ER+ breast cancer tested that are *TP53* wild type, giving the expected outcomes of apoptosis, senescence, and cell cycle arrest, although relative levels of apoptosis are lower than in some other tumour types. The combination of MDM2 inhibition with ERα inhibition emphasises cell cycle and senescence outcomes, rather than apoptosis, which leads to the key question of how p53 activation will perform in the new setting of CDK4/6i resistance in which cells have already overcome a therapeutically enforced cell cycle checkpoint targeted at inducing cell cycle arrest and senescence.

## A Role for MDM2 Inhibition in the CDK4/6i Resistant Setting

As discussed above, the main target of, and major route to resistance to CDK4/6i is the tumour suppressor pRb. Given its central role in the lifecycle of CDK4/6i treatment, a key question is whether inhibition of MDM2 would be a viable strategy in the CDK4/6i resistant setting, and the direct and indirect interactions of pRb with MDM2 and p53 become very relevant. Given the converging and overlapping roles of pRb and p53, it is unsurprising that these two pathways interact across their activity profile. A major target of p53 transcriptional activity is the CDK inhibitor p21 (**Figure 1**), which is one of the main endogenous mechanisms for CDK4/6 inhibition in the cell. In addition to E2F sequestration and G1 cell cycle blockade by hypophosphorylated pRb, p21-mediated inhibition of cell cycle CDKs results in hypophosphorylation of the pocket proteins, pRb paralogues p130 and p107 (47, 48). Hypophosphorylated pocket proteins interact with E2F family proteins to form the dimerization partner, Rb-like, E2F and multi-vulval (DREAM) complex which acts to enforce quiescence upon the cell, keeping it in the G0 arrested state (49, 50). The DREAM complex is a transcriptional repressor and hence *via* p21-mediated inhibition of CDKs is a major contributor to the portion of p53 activity associated with downregulation of cell cycle related transcripts in G1 (distinct from p53 transcriptional activation of e.g. p21 and PUMA) (48). After p53 activation, pRb and the other pocket proteins cooperate to inhibit expression of transcripts associated with progress through G1 and entry to S phase (51). Loss of pRb has been shown in the lab and in the clinic to generate resistance to CDK4/6i but simultaneous knockout of pRb and p130 in fibroblasts rendered cells even more resistant to treatment with the CDK4/6i palbociclib than pRb knockout alone (52). Additionally, p130 and p107, but not pRb, are associated with inhibition of G2/M related transcripts and reduced entry into mitosis following p53 activation (51, 53). Part of the p53 tumour suppressive activity therefore operates *via* p107 and p130 cooperating with pRb to control entry into the cell cycle from G1 (although pRb appears to play the dominant role) and also through p107/p130-mediated cell cycle arrest in G2/M. Consistent with this, in our recent examination of the activity of the MDM2i NVP-CGM097 in ER+ breast cancer models, we were able to observe accumulation of cells in both the G1 and G2/M phases of the cell cycle after p53 activation (29).

In addition to its p53-associated activity towards pRb and other cell cycle control proteins, MDM2 also interacts more directly with these factors to promote both proliferative and anti-proliferative outcomes, depending on context. p53-mediated upregulation of MDM2 protein is a major consequence of p53 activation *via* MDM2 inhibition. However, given that MDM2 is a promiscuous protein interaction partner, it is not currently clear how this pool of MDM2 interacts within the context of the cell, given its potential lack of appropriate interaction partners, expression controls, and post-translational modifications due to its therapeutically induced origin. Whilst the continued presence of MDM2i should prevent the interaction with p53, the effects of increased MDM2 on other interaction partners in the context of MDM2 inhibition remain to be elucidated.

MDM2 interacts directly with pRb (54, 55) and contributes to its degradation (56, 57), independent of its interaction with p53. However, under conditions of genotoxic stress, MDM2 can induce G1 arrest by enhancing translation of Rb mRNA (58), with MDM2 protein chaperoning *RB1* mRNA to the polysomes. This activity requires the DNA damage-dependent phosphorylation of MDM2 so it is unclear how the increased levels of MDM2 produced in response to MDM2i would function in this regard. Phosphorylation of MDM2 is associated with increasingly strong interactions with pRb in cell free assays (54), and increased MDM2-mediated degradation of pRb in G2/M under DNA damage conditions (58). The early generation MDM2i, nutlin-3A caused a dramatic reduction in total pRb levels in several human cancer cell lines (59) and in myoblasts (60). The reduction of pRb levels appeared to be p53-dependent (59), likely as a function of the role for p53 to induce MDM2 transcription. Interestingly, the same study also highlighted differential outcomes for cells dependent upon the effect of MDM2i on pRb phosphorylation. Most cancer cells in the study (including MCF-7 breast cancer cells) responded to nutlin-3A with significant accumulation of hypophosphorylated pRb in a p53/p21 dependent manner and induction of cell cycle arrest. In the SJSA-1 sarcoma line and the LnCAP prostate cancer line, however, little accumulation of hypophosphorylated pRb was observed and rather than enter cell cycle arrest, cells instead became apoptotic. These findings are important from the point of view of using MDM2i to treat CDK4/6i resistant breast cancer where loss or mutation of *RB1* have been identified clinically as mechanisms of CDK4/6i resistance. Given that these data suggest loss of pRb is a reproducible consequence of MDM2 inhibition, tumours harbouring *RB1* deletions or mutations leading to attenuated function as mechanisms of CDK4/6i resistance might therefore still be expected to respond to MDM2i, as long as they retain wildtype *p53*. In fact, the study discussed above (59), and an earlier finding that siRNA-mediated ablation of pRb redirected the cell cycle arrest phenotype in response to Nutlin-3a of HCT116 colon cancer cells towards a robust apoptotic response (61), suggests that MDM2 inhibition may in fact result in higher levels of cytotoxicity in pRb deficient ER+ breast cancer cells compared to the mostly cytostatic effects observed in models to date (29, 46). A study conducted in AML cell lines (62) found that Nutlin-3a treatment resulted in cell cycle arrest for cells in G1 but apoptosis for cells in G2/M, with expression of p21 protecting the cells in G1. This is consistent with the model of cells that accumulate hypophosphorylated pRb entering a cytostatic G1 arrest and cells without this checkpoint proceeding through to G2/M and undergoing apoptosis.

In terms of other mechanisms of CDK4/6i resistance that operate *via* achieving hyperphosphorylation and inactivation of pRb by increased activity of CDK4/6 or dysregulated activity of CDK2, MDM2 inhibition might still be expected to be a viable strategy. In the case that pRb levels are significantly depleted following MDM2 inhibition, mechanisms that have evolved to promote pRb hyperphosphorylation may become redundant. Alternatively, p53-dependent upregulation of the pan-CDK inhibitor p21 that is active against CDK4, 6 and 2 may offer an alternative route to achieve pRb hypophosphorylation and cell cycle arrest.

Given the potential for CDK4/6i resistant cells to be immune to cell cycle arrest in G1, another potential avenue for effective cell suppression by MDM2i might also be arrest in the G2/M cell cycle phase. The role for p53 (and by extension MDM2) in G2/M arrest in coordination with the DREAM complex is discussed above, although this may be susceptible to CDK4/6i resistance mechanisms that promote pRb hyperphosphorylation as the same process would be expected to hyperphosphorylate p107 and p130 leading to the disruption of the DREAM complex. However, it does appear that MDM2 may support the activity of p53/DREAM in suppressing factors that promote progression through G2/M. The mitosis-promoting protein Cdc25C is required for progression through G2/M and is repressed by activation of the DREAM complex (63). Interestingly, Cdc25C was found to be stabilised after MDM2 ablation and was identified as a target for MDM2-mediated degradation (64). In this case, MDM2 and p53 work together to reduce a key cell cycle protein, cooperatively promoting G2/M cell cycle arrest. It is perhaps not surprising, and quite elegant, that the cell would find something useful for all of the MDM2 protein produced by p53 activation to do whilst p53 activity is still required! Interestingly, we recently showed that in a cell line model of palbociclib resistance, treatment with MDM2i did indeed result in a significant accumulation of cells in G2/M but not in G1/S as we had observed in cells sensitive to palbociclib (29).

Although ER+ breast cancer is generally considered to have a relatively low frequency of p53 mutation, these do still occur in around 20% of cases in the primary and metastatic settings (29) and there is some evidence that the frequency of p53 mutation may be enriched in tumours resistant to CDK4/6i, with frequencies between 27% and 58% reported (17, 65, 66). This wide range is potentially driven by the relative levels of prior treatment in the cohorts studied, with reports at the lower end of the range being generated from studies in which patients had received fewer prior lines of therapy on average (17). There is currently no direct evidence for a specific mechanistic role of *TP53* mutation in resistance to CDK4/6i (66). Although MDM2 does perform a variety of p53 independent roles in the cell (67) that could conceivably provide clinical benefit should MDM2 be inhibited, p53 status remains the key determinant of efficacy for MDM2i. On current evidence, one would predict that existing MDM2 targeted drugs would not induce a significant tumour inhibitory response for patients with ER+ breast tumours harbouring mutant *TP53*.

## Potential for Rational Therapeutic Combinations With MDM2i

As discussed above (29, 45, 46), preclinical data for combinations of MDM2i and ER-targeting agents are very encouraging and hence this is a promising area to be explored clinically. In the CDK4/6i resistant setting, there is currently very little preclinical evidence to support particular treatment combinations with MDM2 inhibitors. We recently demonstrated combined activity between NVP-CGM097 and palbociclib in both palbocicilib sensitive and palbociclib resistant cell line models (29). Interestingly, our findings were broadly similar to our observations of the combination of MDM2i with endocrine therapy: the combination treatment promoted an outcome of increased cell cycle arrest and senescence, but not apoptosis, compared to MDM2 inhibition alone in both the sensitive and resistant settings. In contrast, a study using panels of CDK4/6i and MDM2i to treat preclinical models of neuroblastoma (68), and a study that combined the MDM2i nutlin-3A with various CDK4/6i in sarcoma cell lines (69), found no evidence of synergy between MDM2i and CDK4/6i. Both studies reported no increase in the rates of apoptosis, which is similar to the outcome of combining cell cycle inhibitors (either CDK4/6i or endocrine therapy) with MDM2i in ER+ breast cancer models (29, 46), but also no evidence of any combined effect beyond additivity at best. Interestingly, as discussed above, the SJSA-1 cell line used in the sarcoma study (69) has been shown to predominantly respond to MDM2i by way of apoptosis, in contrast to other cell lines in which significant cell cycle arrest and senescence also occurred (59). Given the key role for senescence in synergy between MDM2i and cell cycle targeted therapies in ER+ breast cancer, it is possible that it is the susceptibility of a particular tumour type to senescence that is a key determinant of response to this type of combination therapy. In support of this hypothesis, a recent study that used the combination of MDM2i and CDK4/6i in patient derived models of melanoma with insensitivity or resistance to CDK4/6i (70), did report a synergistic response and identified increased cell cycle arrest and gene expression changes consistent with senescence in combination treated samples. The key mediator of this response was induction of the CDK inhibitor CDKN1A (p21 protein) – a p53 target and mediator of cell cycle arrest and senescence that is also amongst the most strongly upregulated genes in ER+ breast cancer models treated with MDM2i. Clinical trials that combine MDM2i with CDK4/6i have completed phase 1b and the new generation MDM2i siremadlin is currently in phase 2 clinical trials in combination with ribociclib in solid tumours. Alternatively, given the similarity in response between combinations with CDK4/6i and combinations with endocrine therapy, combining MDM2i with endocrine therapy also presents a rational avenue for investigation in the CDK4/6i resistant setting.

Combinations with BH3 mimetics such as venetoclax that target the Bcl-2 family of apoptosis inhibitors offer a potential rational combination in the setting of CDK4/6i resistant ER+ breast cancer. BH3 mimetics are part of a class of drugs known as senolytics which induce apoptosis in a senescent cell population and are of particular interest in treating age-related health issues (71, 72). Bcl-2 itself is a transcriptional target of ER and is upregulated in up to 90% of ER+ breast cancers (73). Other members of the Bcl-2 apoptotic inhibitor class include Bcl-XL and Mcl-1, and small molecule inhibitors of all of these have been trialled clinically (74). Bcl-2 family apoptotic inhibitors are in turn inhibited by BH3-only proteins such as PUMA (75) and NOXA (76) which are transcriptional targets of activated p53 and play a major role in the p53-induced progression to apoptosis. We have recently shown that there is significant regulation of Bcl-2 apoptotic inhibitors in ER+ breast cancer models in response to treatment with MDM2i and endocrine therapy, including upregulation of both Bcl-XL and Mcl-1 that may limit the potential of this treatment combination to induce apoptosis (29). This is consistent with the prevalence of senescence after treatment observed by us and others (29, 46) and offers an opportunity for targeting these senescent cells for apoptosis, either as a MDM2i/BH3 mimetic combination, or perhaps sequential drug treatments. A preclinical study in AML (62) demonstrated significant synergy between nutlin-3a and the Bcl-2/Bcl-XL inhibitor ABT-737. It was found that the drugs were complementary in targeting the cell population for apoptosis with nutlin-3a inducing apoptosis for cells in G2/M but senescence for cells in G1, and ABT-737 targeting the G1/senescent population for apoptosis. Further investigation of the combination of MDM2i and BH3 mimetics in the CDK4/6i resistant setting does therefore present a very rational avenue for further investigation, although as with many MDM2i combinations, overlapping toxicities may be a limiting factor on clinical efficacy.

## MDM2 Inhibitors in Clinical Trials

There is currently limited clinical data on the use of MDM2i in breast cancer. However, a number of agents targeting the p53-MDM2 pathway have been trialled in other malignancies, providing important data on dosing, toxicity profile and clinical activity. The first small molecule MDM2 inhibitor to be synthesised was nutlin-3A (77) and its derivative RG7112, a cis-imidazoline non-genotoxic inhibitor of the p53-MDM2 axis, was the first to be clinically assessed. Although it showed clinical activity in liposarcoma, acute myeloid leukaemia (AML) and chronic lymphocytic leukaemia (CLL), significant gastrointestinal and bone marrow toxicity limited its clinical utility and resulted in the cessation of its development (78, 79). Similarly, NVP-CGM907 was examined in unselected solid tumours (Clinicaltrials.gov identifier NCT01760525) but found to be poorly tolerated due to significant grade 3/4 neutropenia and thrombocytopenia (80). As a result, the development of this drug in solid malignancies was halted.

Development of newer generation of MDM2 inhibitors focused on enhancing potency, selectivity and bioavailability. Idasanutlin (RG7388), which has identical cellular mechanisms to RG7112, was examined in phase 1/1b trials in AML and advanced solid tumours. The trial in advanced solid tumours investigated the optimal schedule, maximum dose tolerated and dose-limiting toxicities of idasanutlin. An optimal dosing schedule of 5 daily dose per 28-day cycle was selected for further development, due to favourable pharmacokinetic and toxicity profile. Best response was stable disease in 30% of patients, with prolonged response seen in 2 patients with sarcoma (81). In contrast with the modest clinical response seen in solid tumours, idasanutlin in AML demonstrated significant responses as both monotherapy and in combination with cytarabine chemotherapy, with a composite complete response rate (cCR) of 29% in the combination arm (82). The phase 3 trial MIRROs trial (Clinicaltrials.gov identifier NCT02545283) of this combination however did not meet its primary end point of prolonging survival (83). Idasanutlin is now being investigated in in a phase 1b/2 trial in combination with the Bcl-2 inhibitor venetoclax in relapsed/refractory AML (Clinicaltrials.gov identifier NCT02670044) based on preclinical studies which demonstrated a synergistic effect (84).

Characterisation of NVP-CGM097 led to the development of a more potent derivative, siremadlin (HDM201). A phase 1 trial of siremadlin in advanced tumours (Clinicaltrials.gov identifier NCT02143635) explored four dose regimens. The most common adverse event was nausea affecting up to 60% of patients but mostly low grade and not dose limiting. The most notable grade 3/4 toxicity was myelosuppression, especially neutropenia and thrombocytopenia. On pharmacokinetic studies, high-dose intermittent regimens reached plasma concentration closer to predicted clinical target efficacious levels required for tumour regression. The dose chosen for the expansion cohort was 120mg D1 and 8, q28 days due to its favourable pharmacokinetic results and low incidence of grade 3/4 thrombocytopenia. The clinical benefit rate of this study was 36%, with 2 patients achieving a partial response (85). Siremadlin was trialled in combination with ribociclib in patients with advanced liposarcoma. In this phase 1b study, toxicities were similar to those of single agent Siremadlin. Dose-limiting toxicities were reported in 16 patients, all except 1 were haematologic. A clinical benefit rate of 53% was observed, with median progression-free survival up to 4.8 months. Siremadlin is currently in trial in combination with ribociclib in advanced solid tumours and trametinib in colorectal cancer (**Table 1**). In haematological malignancies, siremadlin also demonstrated manageable safety and preliminary clinical activity. Overall response rate was 21%, with 3/34 complete responses (94). A phase 1b trial of siremadlin in combination with venetoclax in AML/MDS is currently ongoing (Clinicaltrials.gov identifier NCT03940352).

**Table 1:** MDM2 inhibitors in clinical trials.

Drug name	Clinical Trials identifier *completed	Tumour	Combination treatment	Phase	Response rate(Completed trials only)	Reported grade 3/4 toxicities (Completed trials only)
RG7112	NCT00559533*	Advanced solid tumours		1	26.7% SD (sarcoma subgroup)	Diarrhoea, cytopenia, hyponatremia (86)
	EudraCT 2009-015522*	Liposarcoma (neoadjuvant)		2	5% PR, 70% SD	Neutropenia, thrombocytopenia
	NCT01605526*	Soft tissue sarcoma	Doxorubicin	1	50% SD (interim evaluation)	Neutropenia (60%), thrombocytopenia (45%), febrile neutropenia (22%) (87)
CGM097	NCT01760525*	Advanced solid tumours		1	2.1% PR, 43.8% SD	Thrombocytopenia, neutropenia (80)
Idasanutlin	NCT01462175*	Advanced tumours		1	30.6% SD	Nausea/vomiting, diarrhoea, thrombocytopenia, neutropenia, febrile neutropenia
	NCT03158389	Glioblastoma		1b		
	NCT03555149	Colorectal Cancer	Atezolizumab	1b		
	CRUKE/12/032	Prostate cancer	Abiraterone or enzalutamide	1		
Siremadlin	NCT02143635*	Advanced solid tumours		1	2% PR, 34% SD	Neutropenia, thrombocytopenia, anaemia (85)
	NCT02343172*	Liposarcoma	Ribociclib	1	4% PR, 49% SD	Neutropenia, thrombocytopenia, anaemia (88)
	NCT04116541	Advanced solid tumours	Ribociclib	2		
	NCT02601378	Uveal melanoma	LXS196 (PKC inhibitor)	1		
	NCT03714958	Colorectal Cancer	Trametinib	1		
AMG-232 (KRT-232)	NCT01723020*	Advanced solid tumours		1	No objective response.	Thrombocytopenia, neutropenia (89)
	NCT03787602	Merkel cell carcinoma		2		
	NCT03107780	Glioblastoma		1		
MI-77301	NCT01636479*	Advanced solid tumours		1	Best response SD in 56%	Thrombocytopenia, neutropenia (90)
	NCT01985191*	Advanced solid tumours	Pimasertib	1	4% PR, 63% SD	Thrombocytopenia, fatigue, elevated creatinine, diarrhoea, elevated lipase/amylase, pneumonitis, decreased ejection fraction (91)
APG-115	NCT02935907*	Advanced solid tumours		1	21.4% SD after 2 cycles	Fatigue (10.7%), thrombocytopenia (10.7%) (92)
	NCT03781986	Salivary gland carcinoma	+/- Carboplatin	1b		
BI-907828	NCT03449381	Advanced solid tumours		1		
	NCT03964233	Advanced solid tumours	BI-754091 (anti-PD1)	1		
			BI-754111 (anti-LAG3)			
Milademetan	JapicCTI-142693*	Advanced solid tumours		1	43.8% SD	Thrombocytopenia, anaemia, hyponatremia, transaminitis, neutropenia (93)
	NCT05012397	Advanced solid tumours		2		
ALRN-6924	NCT03725436	Advanced solid tumours	Paclitaxel	1		

*completed.

Several other MDM2 inhibitors have been trialled in early phase clinical trials, mostly in haematological malignancies. AMG-232 (KRT-232) is another oral selective MDM2 inhibitor trialled in phase 1 setting in relapsed/refractory AML, multiple myeloma and advanced solid tumours (89, 95). These demonstrated tolerable safety and preliminary clinical response, leading to several ongoing trials in various haematological malignancies, as well as in Merkel cell carcinoma and glioblastoma. Milademetan demonstrated tolerability and moderate antitumour activity in a phase 1 trial in advanced solid tumours (93). A phase 2 trial is about to commence recruitment (Clinicaltrials.gov identifier NCT05012397). Phase 1 trials in advanced solid tumours are ongoing for several other drugs (**Table 1**).

Ongoing development of MDM2 inhibitors have seen improved safety profile and antitumour activity in newer agents, with increasing potential to be utilised in the clinical setting. One of the major challenges of MDM2 inhibitors has been significant toxicities, predominantly haematological and gastrointestinal. For most agents, myelosuppression is the most common dose-limiting toxicity. This has limited the capacity to combine treatment with other agents, as this typically overlaps with the toxicity profile of most anti-cancer drugs. In the setting of ER-positive breast cancer in combination with hormone-targeting treatment, this challenge may be mitigated, given these treatments do not suppress the bone marrow and rarely causes gastrointestinal side effects. Hence this is a promising area yet to be explored clinically.

## Conclusion

Our current understanding of how MDM2 and p53 interact with ER, pRb and potential mechanisms of endocrine and CDK4/6i resistance do encourage the further preclinical study of MDM2i as novel therapeutics in the CDK4/6i resistant setting. A key area of future research will be the potential combinations of MDM2i with existing and novel therapeutics. One of the major challenges to the use of MDM2i either alone or in combination with other therapies for the treatment of solid tumours such as ER+ breast cancer has been poor tolerability. Ongoing development of MDM2 inhibitors have seen improved safety profiles and antitumour activity in newer agents with increasing potential to be utilised in the clinical setting. Continuing studies to elucidate the new biology of CDK4/6i resistance and how this interacts with MDM2 inhibition will lead to novel strategies to exploit synergies that may lead to more tolerable drug regimens to realise the potential of MDM2i.

## Author Contributions

NP conceived, researched, and wrote the article. JC and EL researched and wrote the article. All authors contributed to the article and approved the submitted version.

## Funding

This work was funded by National Breast Cancer Foundation endowed chair to EL (EC17-002) and the Love Your Sister Foundation.

## Conflict of Interest

NP and EL have received research funding from Novartis Oncology and Bayer pharmaceuticals. EL provides advisory board services to Novartis Australia, Roche Australia, Specialised Therapeutics Australia, Pfizer Australia, Lilly Australia and Amgen Australia (All honoraria paid to the Garvan Institute of Medical Research).

The remaining author declares that the research was conducted in the absence of any commercial or financial relationships that could be construed as a potential conflict of interest.

## Publisher’s Note

All claims expressed in this article are solely those of the authors and do not necessarily represent those of their affiliated organizations, or those of the publisher, the editors and the reviewers. Any product that may be evaluated in this article, or claim that may be made by its manufacturer, is not guaranteed or endorsed by the publisher.
